# Assessment of Knowledge, Attitude and Vaccination Status of Hepatitis B among Nursing Training Students in Ho, Ghana

**DOI:** 10.5334/aogh.750

**Published:** 2019-02-28

**Authors:** Joshua Kwabena Aniaku, Edem Kojo Amedonu, Adam Fusheini

**Affiliations:** 1 Department of Biostatistics and Epidemiology, School of Public Health, University of Health and Allied Sciences, PMB 31, Ho, GH; 2 Department of Health Policy, Planning and Management, School of Public Health, University of Health and Allied Sciences, PMB 31, Ho, GH; 3 Centre for Health Policy/MRC Health Policy Research Group, School of Public Health, University of the Witwatersrand, Private Bag X3, Wits 2050, Johannesburg, ZA

## Abstract

**Background:**

Viral hepatitis B is a disease condition of the liver caused by the hepatitis B virus, and it leads to complications such as cancer and cirrhosis. This poses an occupational hazard because about 66,000 health care workers get infected with the virus annually. Adequate knowledge and right attitude of health workers are required to prevent the disease. Compared to average health care workers, trainee nurses are more vulnerable to the disease due to inadequate knowledge on infection control guidelines.

**Purpose of the Study:**

The study assessed the knowledge, attitude and vaccination status of hepatitis B among nursing training students in Ho, Ghana.

**Method:**

A descriptive cross-sectional study was carried out between September and December 2017 in which 358 student nurses were randomly selected to participate in this study. A self-administered structured questionnaire was used as a data collection tool to get information from the students. Data were then entered into SPSS version 20 for cleaning and analysis.

**Result:**

The majority of participants were between the ages of 20–26 years with the mean age 21.56 (SD ± 2.65). About 78.2% knew the disease is caused by a virus. Also, 69.8% reported transmission through needle stick injuries, and the mean knowledge score was 29.6 (SD ± 6.98). Also, 68.8% recapped needles (P = 0.012), and 49.4% have taken the full three doses of vaccines.

**Conclusion:**

The study points to a satisfactory knowledge and vaccination status of hepatitis B among the trainee nurses but poor attitude toward the disease, hence the need for massive health education among the nurses.

## Introduction

Viral hepatitis B is a disease condition of the liver caused by the hepatitis B virus from the Hepadnaviridae family of viruses. Hepatitis B is a highly infectious disease that inflames the liver and eventually leads to complications such as liver damage, liver cancer and cirrhosis. The disease has both acute and chronic phases, with the acute phase being a new infection. After six months of persistence, the acute phase often results in chronic infection, which lasts a lifetime [1]. The major mode of transmission is through contact with blood, or other body fluids of an infected person [1]. Other modes of transmission include the use of contaminated razors or toothbrushes, needles, syringes or other drug-injection equipment from an infected person. Also, direct contact with open sores of an infected person and exposure to blood from needle sticks or other sharp instruments from an infected person can lead to transmission of the virus [2]. Thus, everyone is at risk of the disease.

Hepatitis B virus was discovered in 1963, and the vaccine against the disease was also discovered in 1965, by Dr. Baruch Blumberg and his colleagues [3]. The disease is still an important public health problem with 240 million people chronically infected (defined as hepatitis B surface antigen positive for at least six months) globally and more than 686,000 people dying every year due to complications, the early discovery of the vaccine notwithstanding.

Sub-Saharan Africa and East Asia has the highest prevalence with 5–10% of the adult population chronically infected [1]. In Ghana, the prevalence rate of the disease is 12.3% [4], making it one of the highest globally and underscoring the need for this study. Ghana has made strides to reduce mortality and morbidity of the disease by combining the hepatitis B vaccine with DPT and Hib vaccines to form a pentavalent vaccine. This was introduced in the country’s expanded program on immunization (EPI) in the year 2002. Three doses of the pentavalent vaccine are inoculated into children under five years at 6 weeks, 10 weeks and 14 weeks after birth. The government of Ghana bears the cost of the vaccination for this category of children. Individuals above this age can also take the vaccination within a period of three months at their own cost. The introduction of the vaccine has seen a relative decline of the disease. However, the problem still persists. A number of factors such as late introduction of the vaccine into the country’s expanded program on immunization (EPI), cost of the vaccine for adults, lack of awareness, poverty and reticence to change [5] have been identified as explanation. Besides, the disease receives less funds and attention by policy makers in the country despite the fact that it is more infectious than HIV/AIDS contributing to its persistent prevalence in the country [2].

The high prevalence and mode of spread of this infectious disease in Ghana makes it an occupational hazard to all health workers and trainee students in particular who work in hospitals and health facilities. Throughout the world, millions of healthcare professionals work in health institutions and it is estimated that 600,000 to 800,000 experience cut and puncture injuries occurring among them per year, of which approximately 50% are not registered. The annual proportion of healthcare workers exposed to blood-borne pathogens was 5.9% for hepatitis B, corresponding to about 66,000 hepatitis B virus infections in health-care workers worldwide [6]. This makes trainee nurses more vulnerable to the disease since they lack the requisite knowledge and competence expected of health workers for them to be protected. This underscores the need for them to have adequate knowledge regarding the disease because it is assumed that adequate knowledge of the disease will in turn influence their attitude toward the disease and subsequently impact their vaccination status. Scholars observed that in relation to the disease, there exists a gap between knowledge and practice because many healthcare workers are still not immunized against hepatitis B [7]. Again, a study conducted among medical and health science students established that 4.7% of the study participants completed all three doses of their vaccination schedule; 8.7% students were incompletely vaccinated. Lack of information in 20.8% of the students, no need by 2.8% students, fear of injection by 4.7% and 14% ignorance [8] were attributable factors for not vaccinating. A study conducted among health care workers in Bantama, Ghana, also highlighted unsatisfactory or poor knowledge, attitude and practice toward hepatitis B virus and some important aspects of viral hepatitis [8]. One-quarter of the study participants had been exposed to “needle stick injury,” but more than half of them still showed negative attitude toward testing after exposure. From the above, health students need to have adequate knowledge of hepatitis B right from school because the absence or lack of this knowledge will eventually influence their attitude and lifestyle when they become workers. This study assessed knowledge, attitude and vaccination status of trainee nurses regarding viral hepatitis B.

## Methodology

A descriptive cross-sectional study was carried out between September and December 2017 on Nurses Training College (NTC) campus in the Ho Municipality of the Volta Region of Ghana. The campus is located in Medical Village and shares boundaries with the Volta Regional Hospital and Mawuli Senior High School. The school has an estimated student population of about 2,100 and runs the following programs: Registered General Nursing (RGN), Registered Community Nursing (RCN) and Registered Nurse Assistant, Preventive (RNAP). Using single proportion-based statistical formula with estimated prevalence of 50%, margin of error 5%, confidence interval of 95% and non-response rate of 10%, the minimum sample size was 358. Stratified and simple random sampling techniques were used to ensure representativeness. The strata were based on program and year of study. Therefore, seven strata were used, first to third year: RGN, RCN and RNAP. However, there were no third years in the RNAP program because it is a two-year program. Simple random sampling was used to draw participants proportionately from each stratum. The data collection tool used was self-administered, close-ended, structured questionnaires to gather information from the students. Data collected were entered into IBM SPSS Statistics Version 20 for cleaning and analysis. Descriptive statistics, frequency, percentages, mean and standard deviation were used to summarize data. Vaccination status was measured using the number of trainees who reported being vaccinated as the numerator and the total trainees who responded to the question as the denominator. Knowledge was assessed using a composite variable of 18 items and trichotomized into three (very good, good and poor knowledge). A correct answer was assigned the code “1.” Wrong answers were assigned the code “2.” The responses were computed and categorized as follows: good knowledge, 18 to 24; average knowledge, 25 to 30; and poor knowledge, 31 to 36. On the other hand, attitude was measured using 14 items. Chi square analysis was employed to determine the association between knowledge of the trainees regarding hepatitis B infections and their attitude toward the infection. A p-value less than 5% was considered statistically significant. Ethical clearance was obtained from the Ghana Health Service Ethical Review Committee with approval number GHS-ERC: 25/05/17 before commencement of the research. Students who gave their consent in written form were included in the study.

## Results

A total of 358 student nurses gave responses to the results generated for the study. Females were in the majority, being 73.5% of the total participants. For age of participants, the majority were between the ages of 20–26 years with mean age of 21.56 (SD ± 2.65). Also, representation of students by year of study was 41.6%, 38.3% and 20.1% for first, second and third year, respectively (Table 1).

**Table 1:** Demographic Characteristics of Participants.

Variables	Frequency	Percentage (%)

**Age group**		
17–19	66	21.6
20–26	225	73.8
27–35	14	4.5

**Sex**		
Males	95	26.5
Females	263	73.5

**Religion**		
Christianity	348	97.2
Islam	7	2.0
Others	3	0.8

**Ethnicity**		
Ewe	232	64.8
Ga	28	7.8
Akan	66	18.4
Others	32	8.9

**Marital status**		
Single	341	95.3
Married	8	2.2
Cohabiting	9	2.5

**Employment status**		
Employed	25	7.0
Unemployed	333	93.0

**Region of residence**		
Volta	244	68.2
Accra	66	18.4
Eastern	31	8.7
Western	3	0.8
Central	9	2.5
Northern	1	0.3
Ashanti	4	1.1

**Address**		
Urban	268	74.9
Rural	90	25.1

**Program of study**		
RGN	183	51.1
RCN	111	31.1
NAP	64	17.9

**Level of study**		
1st year	149	41.6
2nd year	137	38.3
3rd year	72	20.1

For distribution of knowledge on hepatitis B, 97.8% of participants had heard about hepatitis B. The majority of participants (78.2%) knew that the disease is caused by a virus. Participants also reported that hepatitis B can be transmitted through a number of ways, 65.6% said it can be transmitted through sex and 79.6% through blood transfusion. Also, 69.8% were aware the disease can be gotten through needle stick injuries, and 57.8% said through child birth. Furthermore, 76.3% knew the disease is more infectious than HIV/AIDS, and 51.4% said it was curable. About 70.4% reported the hepatitis B virus causes liver inflammation, and as low as 46.9% reported jaundice to be a symptom of the disease. The overall mean knowledge score of participants is 29.6 (SD ± 6.98). See Table 2.

**Table 2:** Distribution of Participants’ Knowledge on Hepatitis B Virus.

Variables	Frequencies	Percentages

**Heard of Hep B**		
Yes	350	97.8
No	8	2.2

**Source**		

**Print media**		
Yes	18	5
No	340	95
**School**		
Yes	217	60.6
No	141	39.4
**Radio/TV**		
Yes	72	20.1
No	286	79.9
**Friends/Neighbor**		
Yes	14	3.9
No	344	96.1
**Health worker**		
Yes	78	21.8
No	280	78.2

**Cause**		
Bacteria	17	4.7
Virus	280	78.2
Fungi	5	1.4
Don’t know	56	15.6

**Transmission routes**		

**Sex**		
Yes	235	65.6
No	68	19.0
Don’t know	55	15.4

**Blood transfusion**		
Yes	285	79.6
No	35	9.8
Don’t know	38	10.6

**Sharing of towel**		
Yes	177	49.4
No	101	28.2
Don’t know	80	22.3

**Air**		
Yes	68	19.0
No	224	65.6
Don’t know	66	18.4

**Faeco-oral**		
Yes	145	40.5
No	88	24.6
Don’t know	125	34.9

**Needle stick injury**		
Yes	250	69.8
No	30	8.4
Don’t know	78	21.8

**Child birth**		
Yes	207	57.8
No	53	14.8
Don’t know	98	27.4

**Holding hands**		
Yes	99	27.7
No	203	56.7
Don’t know	56	15.6

**More infectious than HIV/AIDS**		
Yes	273	76.3
No	33	9.2
Don’t know	52	14.5

**Asymptomatic at acute phase**		
Yes	114	31.8
No	69	19.3
Don’t know	175	48.9

**Jaundice a symptom**		
Yes	168	46.9
No	50	14.0
Don’t know	140	39.1

**Affects other organs**		
Yes	162	45.3
No	73	20.4
Don’t know	123	34.4

**Cirrhosis and liver cancer**		
Yes	181	50.6
No	12	3.4
Don’t know	165	46.1

**Liver inflammation**		
Yes	252	70.4
No	12	3.4
Don’t know	94	26.3

**Carriers as risk**		
Yes	301	84.1
No	19	5.3
Don’t know	38	10.6

**Curability**		
Yes	184	51.4
No	110	30.7
Don’t know	64	17.9

In cross tabulating knowledge score and demographic characteristics, there was significant association between knowledge score and the characteristics age and level of study. Participants having average (57.1%) and good (42.9%) knowledge were between the ages 27–35. The majority (53%) of participants in age group 17–19 had poor knowledge (P = 0.001). Also, most of the participants fell within the average knowledge category: first years 104 (69.8%), second years 103 (75.2%) and third years 31 (43.1%). The majority of those in their third year (52.8%) had good knowledge about the disease (P = 0.000). Refer to Table 3.

**Table 3:** Association of Knowledge with Selected Socio Demographic Variables.

Variables	Knowledge Score			Chi value (x^2^)	P-value

	Good, n (%)	Average, n (%)	Poor, n (%)		

**Age group**					
<19	35 (53.0)	20 (30.3)	11 (16.7)		
20–26	71 (31.6)	86 (38.2)	68 (30.2)	18.809	**0.001**
>27	0 (0.0)	8 (57.1)	6 (42.9)		

**Sex**					
Males	25 (26.3)	57 (60.0)	13 (13.7)	4.9183	**0.086**
Females	42 (16.0)	181 (68.8)	40 (15.2)		

**Religion**					
Christianity	65 (18.7)	232 (66.7)	51 (14.7)		
Islam	2 (28.6)	13(42.9)	2 (28.6)	3.3670	0.498
Others	0 (0.00)	3 (100.0)	0 (0.0)		

**Ethnicity**					
Ewe	40 (17.2)	155 (66.8)	37 (16.0)		
Ga	5 (17.9)	17 (60.7)	6 (21.4)	3.7998	0.704
Akan	15 (22.7)	45 (68.2)	6 (9.1)		
Others	7 (21.9)	21 (65.6)	4 (12.5)		

**Marital status**					
Single	65 (19.0)	225 (66.0)	51 (15.0)		
Married	0 (0.00)	7 (87.5)	1 (12.5)	2.2396	0.692
Cohabiting	2 (22.2)	6 (66.7)	1 (11.1)		

**Employment status**					
Employed	3 (12.0)	19 (76.0)	3 (12.0)	1.1567	0.561
Unemployed	64 (19.2)	219 (65.8)	50 (15.0)		

**Region of residence**					
Volta	50 (20.5)	160 (65.6)	34 (13.9)		
Accra	9 (13.6)	48 (72.7)	9 (13.6)		
Eastern	5 (16.1)	18 (58.1)	8 (25.8)	7.2534	0.840
Western	1 (33.3)	2 (66.7)	2 (66.7)		
Central	1 (11.1)	7 (77.8)	1 (11.1)		
Northern	0 (0.0)	1 (100.0)	0 (0.0)		
Ashanti	1 (25.0)	2 (50.0)	1 (25.0)		

**Address**					
Urban	47 (17.5)	179 (66.8)	42 (15.7)	1.3471	0.510
Rural	20 (22.2)	59 (65.6)	11 (12.2)		

**Program of study**					
RGN	35 (19.1)	114 (62.3)	34 (18.6)		
RCN	19 (17.1)	82 (73.9)	10 (9.0)	5.9605	0.202
NAP	13 (20.3)	42 (65.6)	9 (14.1)		

**Level of study**					
1st yea^r^	9 (6.0)	104 (69.8)	36 (24.2)		
2nd year	20 (14.6)	103 (75.2)	14 (10.2)	82.6787	**0.000**
3r^d^ year	38 (52.8)	31 (43.1)	3 (4.2)		

For participants’ attitude toward hepatitis B, 83.3% reported they always used sterile syringes, and 75.8% used sterile gloves. Also, 68.8% recapped needles after use, and 26.5% had had needle prick injuries. It was good to know that 71.7% acknowledged that control guidelines can help prevent nurses from getting the disease. Though 94.2% of participants were willing to test for the disease, 50.3% said the hepatitis B vaccine was costly. Only 40.5% of participants perceived the disease as a great risk to them (Table 4).

**Table 4:** Distribution of Participants’ Attitudes Toward Hepatitis B.

Variables	Frequencies	Percentages

**Multiple sex partners**		
Yes	33	10.8
No	272	89.2

**Unprotected sex**		
Yes	72	23.8
No	231	76.2

**Use sterile syringe**		
Always	179	83.3
Sometimes	19	8.8
Don’t remember	17	7.9

**Use sterile gloves**		
Yes	163	75.8
No	52	24.2

**Recapped needles**		
Yes	148	68.8
No	67	31.2

**Needle prick injuries**		
Yes	57	26.5
No	158	73.5

**Splash blood or body fluid on skin**		
Yes	23	10.6
No	171	79.7
Don’t know	21	9.7

**Tested after splash and needle prick injury**		
Yes	21	26.9
No	57	73.1

**Control guidelines protects one from HBV**		
Yes	215	71.7
No	24	8.00
Not sure	61	20.3

**Vaccine expensive**		
Yes	168	50.3
No	128	38.3
Don’t know	38	11.4

**Willing to test for HBV**		
Yes	306	94.2
No	19	5.9

**Self-perceived risk of HBV**		
No risk	84	21.2
Risk	205	57.3
Don’t know	77	21.5

For association of knowledge with attitude, 27.9% of participants demonstrated good knowledge, whereas 64.3% with average knowledge reported always using a sterile syringe when attending to patients. It was interesting to note that participants’ knowledge could not deter them from the practice of recapping needles because 33.1% of respondents with good knowledge and 58.1% with average knowledge (P = 0.012) recapped needles. It was also evident that knowledge influenced participants’ perception on control guidelines because 12.6% of respondents with poor knowledge and 22.8% with good knowledge responded that control guidelines within the hospital can help prevent the disease among workers (P = 0.054). Also, 11.9% of respondents with poor knowledge, 67.9% with average knowledge and 20.2% with good knowledge said the hepatitis B vaccine was expensive (P = 0.010). Again, knowledge influenced self-perceived risk of the disease among respondents: 12.7% with poor knowledge, 65.4% with average knowledge and 22.0% with good knowledge responded that the disease posed great risk to them (P = 0.051), as shown in Table 5.

**Table 5:** Association of Knowledge with Attitudes.

Variables	Knowledge Score			Chi value (x^2^)	P-value

	Good, n (%)	Average, n (%)	Poor, n (%)		

**Multiple sex partners**					
Yes	9 (27.3)	22 (66.7)	2 (6.1)	2.6840	0.261
No	51 (18.8)	181 (66.5)	40 (14.7)		

**Unprotected sex**					
Yes	20 (27.8)	45 (62.5)	7 (9.7)	4.4108	0.110
No	40 (17.3)	156 (67.5)	35 (15.2)		

**Use sterile syringe**					
Always	50 (27.9)	115 (64.3)	14 (7.8)		
Sometimes	8 (42.1)	10 (52.6)	1 (5.3)	4.4243	0.352
Don’t remember	2 (11.8)	4 (82.4)	1 (5.9)		

**Use sterile gloves**					
Yes	46 (28.2)	106 (65.0)	11 (6.8)	0.4742	0.789
No	14 (26.9)	33 (63.5)	5 (9.6)		

**Recapped needles**					
Yes	49 (33.1)	86 (58.1)	13 (8.8)	8.8978	**0.012**
No	11 (16.4)	53 (79.1)	3 (4.5)		

**Needle prick injuries**					
Yes	16 (28.1)	37 (64.9)	22 (7.0)	0.0203	0.990
No	44 (27.9)	102 (64.6)	12 (7.6)		

**Splash blood or body fluid on skin**					
Yes	6 (26.1)	14 (60.9)	3 (13.0)		
No	50 (28.9)	111 (64.2)	12 (6.9)	7.6245	0.471
Don’t know	5 (23.8)	15 (71.4)	1 (4.8)		

**Tested after splash and needle prick injury**					
Yes	6 (28.6)	15 (71.4)	0 (0.00)	3.0439	0.218
No	17 (29.8)	33 (57.9)	7 (12.2)		

**Control guidelines protects one from**					
Yes	49 (22.8)	139 (64.7)	27 (12.6)		
No	6 (25.0)	17 (70.8)	1 (4.2)	9.3008	**0.054**
Not sure	5 (8.2)	44 (72.1)	12 (19.7)		

**Vaccine expensive**					
Yes	34 (20.2)	114 (67.9)	20 (11.9)		
No	28 (21.9)	86 (67.12)	14 (10.9)	13.2234	**0.010**
Don’t know	3 (7.9)	23 (60.5)	12 (31.6)		

**Willing to test for HBV**					
Yes	60 (19.6)	204 (66.7)	42 (13.7)	0.6656	0.717
No	5 (26.3)	11 (57.9)	3 (15.8)		

**Self-perceived risk of HBV**					
No risk	16 (19.0)	52 (61.9)	16 (19.0)		
Risk	45 (22.0)	134 (65.4)	26 (12.7)	15.4601	**0.051**
Don’t know	6 (7.8)	52 (67.5)	19 (24.7)		

**Figure 1:**
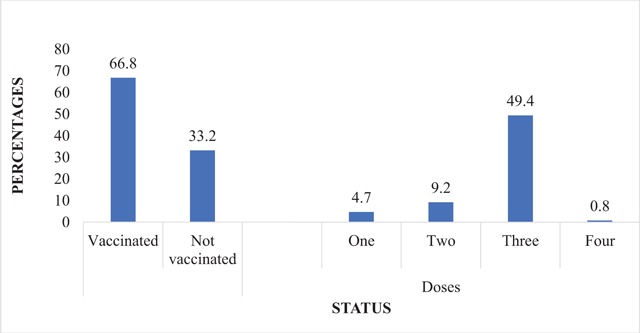
Vaccination Status of Hepatitis B among Participants.

Finally, for vaccination status, 60.1% have tested for hepatitis B, 66.8% of respondents have been vaccinated and 49.4% of those vaccinated reported having taken the full dose of the vaccine. There was significant association between program of study and vaccination status. For program of study, 59.3% of general nurse students have been vaccinated, but a low proportion of students specializing in the specific aspect of nursing had vaccinated: 28.1% of community nurses and 12.6% of nursing assistants (P = 0.000). There was also a significant association between vaccination status and year of study because 29.9% of first years, 46.3% of second years and 23.8 of % third years have vaccinated (P = 0.000). It was further observed that knowledge influenced participants’ vaccination status: 75.8% of participants with good knowledge have been vaccinated against the disease (P = 0.067) with 62.7% also reporting to have completed the three doses (Figure 1).

## Discussion

Healthcare workers are at risk of exposure and possible transmission and infection of hepatitis B because a majority of them are in constant contact with patients or infective material from patients [9]. Knowledge acquired and attitude employed at the workplace are key to preventing one from getting nosocomial diseases. Overall knowledge about hepatitis B was satisfactory, though there were some unsatisfactory responses from participants on hepatitis B transmission modes. Although attitude toward the disease was poor, vaccination status was satisfactory.

In this study, age group and level of study were significant demographic characteristics that had an association with participants’ knowledge on hepatitis B. Majority of participants were within the ages of 20–26 years, and the mean age was 21.56 (SD ± 2.65) years. This is consistent with a number of studies carried out in other parts of the world (P = 0.001). A study among nursing students in Nepal had a slightly lower mean age of 18.54 years (±SD 2.001), with the majority within the age group 18–20 years [10]. For level of study, a majority of participants with very good knowledge 61.1% were in their third year of study as against first-year students 57.0% with poor knowledge about the disease (P = 0.000). This finding is consistent with an earlier study among medical students, which found first years to have poor knowledge and lack of awareness about hepatitis B, its routes of transmission, risk factors and modes of preventions compared to the fifth-year medical students [11]. The suggestion is that adequate knowledge about the disease among the third years could be due to knowledge gained from exposure to tuitions on disease transmission modes and prevention because they have stayed longer in school compared to first years [11].

Knowledge of participants in this study was satisfactory, with 59.5% having the right knowledge about hepatitis B transmission routes and prevention. Though this was satisfactory, it was lower than a study from India among medical interns, which had 83.3% of participants having adequate knowledge, but it was higher than another study in Iraq that recorded a low proportion (14%) of study participants having good knowledge [12]. In this study, 78.2% of participants correctly responded that the disease was caused by a virus. This is a high knowledge about the causative organism but is relatively lower than a study by Paudel and Prajapati, where about 92.2% had good understanding about the causative agent of hepatitis B. Though 84.1% of participants knew that carriers of hepatitis B are at risk of infecting others, surprisingly, about half of participants do not know that the disease leads to cirrhosis and hepatocarcinoma [13]. Again, 48.9% did not know whether hepatitis B was asymptomatic at the acute phase. This is dangerous because knowledge of the dangers hepatitis B poses to these nurses can play an essential role by influencing their preventive attitude against the disease. Also, the study found 69.8% to show adequate knowledge that the disease can be transmitted through needle stick injuries. In a study among medical students in Saudi Arabia, 92.4% of respondents were reported to have said the disease is transmissible through needle stick injury. Furthermore, as high as 23.7% did not know the disease is more infectious than HIV/AIDS [13] as against a study among health workers in Ethiopia that found only 5.0% not knowing. This disparity could be due to a significant difference in length of experience between the students and the health workers [14]. It was also disturbing to notice more than half of respondents acknowledging that the disease is curable. A study among health workers in Nigeria also recorded 62.1% of the workers saying the disease was curable. In Ghana, nurses are sources of health information, including trainee nurses. High wrong response about the curability means high possibility of wrong information delivered to the public.

In this study, though knowledge about the disease was satisfactory, attitude toward the disease was not satisfactory. This is similar to two other studies, where practices were poor in spite of participants showing good knowledge on the disease and its preventive measures [8,15]. Though 71.7% responded that following control guidelines can prevent one from getting the disease, about 27.0% of participants reported they have had needle stick injury (P = 0.000). This is 20% higher than a study by Hussain, Ahmad and Muslehuddin, where only 7.0% of students had needle injury [16]. Also, 73.1% who had needle stick injury and blood splashes did not test for hepatitis B afterward, and that may be due to a majority of students being unaware of the proper action after these accidents [13]. Bhattarai, Kc, Pradhan, Lama, and Rijal found recapping leading to 19.0% of injuries in their study [17]. As high as 68.8% also recapped needle after use in this study, which is better than 73.4% from a study among Iranian medical specialists and could be the reason for the high needle stick injuries recorded [18]. Recapping can make nurses miss the cap and stab their hands leading to infections. It was also noticed that 16.6% and 24.2% use sterile syringes and sterile gloves, respectively, when handling potential infectious sources (P = 0.031 and P = 0.004, respectively). Mesfin and Kibret recorded similar results where 16.5% did not use sterile syringes, and 27.0% of the respondents did not use any gloves while handling surgical instruments [19] Changing of gloves during routine medical proceedings could be deemed a waste of time [15]. A study among medical students reported 16.0% and 6.4% not knowing what to do and disagree, respectively, whether to use gloves when treating a bleeding person with hepatitis B [20]. Nurses are at risk of contracting hepatitis B, but only 57.0% of participants responded to being at risk, and surprisingly only 29.0% of participants with very good knowledge responded to being at risk of the disease.

Vaccination status among the participants was also satisfactory, where about 60% have undergone screening for hepatitis B, 66.8% being vaccinated and 49.4% of those vaccinated reporting to have taken the full three doses of the vaccine. Though the vaccination status was satisfactory, it was still lower compared to 79.0% of medical students in Pakistan who reported they were vaccinated, and 70.6% of them were completely vaccinated [21]. A limitation of this study was non-representativeness of the third years because the NAP program is run for two years in the school. Other limitations of the study were lack of responses from participants on some of the questions posed to them. Although these limitations exist, this study serves as a reference on the first assessment of knowledge, attitude and vaccination of trainee nurses in Ghana.

## Conclusion and Recommendations

The study found the knowledge and vaccination status among trainee nurses to be satisfactory but attitude toward the disease to be poor. Though the knowledge was satisfactory, some nurses were unable to answer some questions on the modes of transmission and the curability of the disease satisfactorily. Ghana’s training nursing schools produce general and community nurses who serve in tertiary, secondary and primary health facilities with the aim of dealing with individuals at the community level. They are responsible for caring for patients and bringing health education to the doorstep of individuals at health facilities, school children and the community at large. A deficit in knowledge regarding hepatitis B will lead to massive misinformation, which can be fatal to a country with hepatitis B prevalence as high as 12.3%. This then calls for a massive health education and a review of the teaching curriculum among these nurses. The training institutions can use the World Hepatitis B Day, which is celebrated every year, to embark on campaigns with the aim of creating hepatitis B awareness among the nurses as well.

Knowledge about hepatitis B alone cannot prevent the disease. The right knowledge reflecting in the attitude of trainee nurses will help prevent infection of the disease among them. Currently, there exists a gap between knowledge and attitude among the students. Nursing institutions should develop courses on Universal Work Precautions, which should be taught within the first and subsequent years of study in the schools. Senior nurses who practically train these students during their clinicals must be given the mandate of ensuring these precautions are observed totally.

Finally, nurses are at high risk of the disease, and they should be fully immunized. However, that is not the case. Ghana’s Ministry of Health should formulate a policy ensuring 100% hepatitis B vaccination coverage in nursing training institutions. This can be achieved by subsidizing the cost of the vaccine and making it a requirement for admission to the various institutions. This will also go a long way in preventing the spread of hepatitis B.
